# Efficacy and Safety of Drug Combinations for Chronic Pelvic Pain: Protocol for a Systematic Review

**DOI:** 10.2196/21909

**Published:** 2021-05-17

**Authors:** Mohammed Mohiuddin, Rex Park, Ursula Wesselmann, Caroline Pukall, Keith Jarvi, Curtis Nickel, Christopher Doiron, Ian Gilron

**Affiliations:** 1 Department of Anesthesiology & Perioperative Medicine Kingston General Hospital Queen’s University Kingston, ON Canada; 2 Departments of Anesthesiology and Perioperative Medicine Division of Pain Medicine, Neurology and Psychology The University of Alabama at Birmingham Birmingham, AL United States; 3 Department of Psychology Centre for Neuroscience Studies, School of Rehabilitation Therapy Queen's University Kingston, ON Canada; 4 Division of Urology Department of Surgery University of Toronto Toronto, ON Canada; 5 Department of Urology Queen's University Kingston, ON Canada

**Keywords:** chronic pelvic pain, combination therapy, clinical studies, systematic review, chronic prostatitis, chronic pelvic pain syndrome, CPPS, interstitial cystitis, bladder pain syndrome, efficacy, safety, drug, pain, pelvis, chronic pain, protocol, therapy, treatment, combination

## Abstract

**Background:**

Chronic pelvic pain with various etiologies and mechanisms affects men and women and is a major challenge. Monotherapy is often unsuccessful for chronic pelvic pain, and combinations of different classes of medications are frequently prescribed, with the expectation of improved outcomes. Although a number of combination trials for chronic pelvic pain have been reported, we are not aware of any systematic reviews of the available evidence on combination drug therapy for chronic pelvic pain.

**Objective:**

We have developed a protocol for a systematic review to evaluate available evidence of the efficacy and safety of drug combinations for chronic pelvic pain.

**Methods:**

This systematic review will involve a detailed search of randomized controlled trials investigating drug combinations to treat chronic pelvic pain in adults. The databases searched will include the Cochrane Central Register of Controlled Trials (CENTRAL), MEDLINE, and EMBASE from their inception until the date the searches are run to identify relevant studies. The primary outcome will be pain relief measured using validated scoring tools. Secondary outcomes, where reported, will include the following: adverse events, serious adverse events, sexual function, quality of life, and depression and anxiety. Methodological quality of each included study will be assessed using the Cochrane Risk of Bias Tool.

**Results:**

The systematic review defined by this protocol is expected to synthesize available good quality evidence on combination drug therapy in chronic pelvic pain, which may help guide future research and treatment choices for patients and their health care providers.

**Conclusions:**

This review will provide a clearer understanding of the efficacy and safety of combination pharmacological therapy for chronic pelvic pain.

**Trial Registration:**

PROSPERO International Prospective Register of Systematic Reviews CRD42020192231; https://www.crd.york.ac.uk/prospero/display_record.php?RecordID=192231

**International Registered Report Identifier (IRRID):**

PRR1-10.2196/21909

## Introduction

### Description of the Condition

Chronic pelvic pain is a highly prevalent condition affecting both men and women. Similar to other chronic pain disorders, it is poorly understood and is associated with various etiologies, broad definitions, treatments that are mostly empirical, and unsatisfactory patient outcomes [1]. There are three frequently studied pelvic pain disorders: interstitial cystitis/bladder pain syndrome (IC/BPS) in men and women, chronic prostatitis/chronic pelvic pain syndrome (CP/CPPS) in men, and chronic pelvic pain (CPP) in women [1].

CPP in women is the most common reason for referral to women’s health specialists, accounting for up to 20% of all outpatient appointments in secondary care [2]. In primary care, it has an incidence of 38 per 1000 women, comparable to back pain (41 per 1000). CPP has global prevalence rates ranging from 2.1% to 24% [2], with estimates as high as 14.7% and 25% in the United States and United Kingdom, respectively [3-5]. It accounts for 1 in 10 outpatient gynecological visits [6], and it is the indication for 40% of laparoscopies and 15% of hysterectomies in the United States [7,8]. It is also associated with significant costs to the health care system, with US $881.5 million per year being devoted in the United States [4], and £158 million in the United Kingdom [9]. The focus of CPP of various etiologies can be experienced in anatomic locations such as the pelvis, anterior abdominal wall, lower back or buttocks, or genitalia (vulva and/or vagina) and can frequently lead to limitations in daily home life and activities [10], including sexual dysfunction. Furthermore, it often requires medical treatment, and can be responsible for a significant amount of missed work [4]. There are numerous proposed causes of CPP [10] involving the gynecologic, urologic, gastrointestinal, musculoskeletal, and neurologic systems, although their pathophysiologic mechanisms are not well understood. Furthermore, for a large proportion of patients, there is no identifiable cause of pain, even after laparoscopic investigation [8,11]. Consequently, CPP presents a great challenge to patients and clinicians, leading to unsatisfactory treatment with minimal pain relief [12,13].

CP/CPPS is the most common urological disorder for men under 50 years and is the third most common diagnosis for men over 50 [14]. One study following 1310 patients across 16 years found an average age of 45 years [15]. It is estimated that up to 10% of males will experience symptoms of CP/CPPS at some point in their lifetime. The global prevalence of prostatitis is estimated to be around 7.1%. It constitutes 1% of primary care visits and 8% of urology consultations in the United States [16], affecting men of all ages and ethnicities, with a mean age of onset of 42 years [14]. The National Institutes of Health (NIH) classification categorized four categories of prostatitis, with the noninfectious type—Category III (chronic nonbacterial prostatitis/chronic pelvic pain syndrome)—being the most common form of symptomatic prostatitis, making up around 90%-95% of prostatitis diagnoses [17]. The two main clinical features of CP/CPPS are pelvic pain and lower urinary tract symptoms (LUTS) [17]. Patients typically report pain in the perineum, penis, testicles, scrotum, or suprapubic region, which can be exacerbated on ejaculation [18]. Common urinary symptoms include storage and voiding LUTS. Consequently, the syndrome is associated with significant decrease in quality of life and in many cases sexual dysfunction [15,17]. Similar to CPP in women, there are various pathophysiological mechanisms proposed, such as infection [19], inflammation [20], neurologic, psychosocial, neuropathic, muscle dysfunction, and psychiatric factors [21-23].

IC/BPS has an estimated prevalence of 3%-7% in women and 2%-4% in men, translating to over 10 million affected individuals in the United States [24,25]. Approximately 90% of patients are female, and 95% of patients have a median age of 40 years [24]. Interestingly, of men who have IC/BPS, about 17% have been found to also have CP/CPPS [25]. Furthermore, a significant proportion of patients have also been found to have conditions such as irritable bowel syndrome, fibromyalgia, and chronic fatigue syndrome, which can add complexities to management of this condition [26]. Patients with IC/BPS frequently experience pain or discomfort perceived to be related to the urinary bladder, and lower urinary tract symptoms such urinary urgency, frequency, and dysuria [27]. They also experience significant feelings of helplessness, depression, lack of control, and catastrophizing, further reducing the quality of life for these patients [28]. Despite its wide prevalence, the majority of pharmacological interventions used are off-label [27], and have scarce or contradictory evidence supporting them [27,29-34]. One study of the literature found a disconnect between guidelines for treatment and the actual effectiveness in clinical practice, indicating the need for integrating evidence from a variety of potential treatments [35].

### Description of the Intervention

As there are numerous contributing factors to chronic pain in the pelvic region, several interventions have been proposed for its treatment, with diverse mechanisms of action. These include α-blockers, 5-α-reductase inhibitors, quinolones/tetracyclines, phytotherapy, nonsteroidal anti-inflammatory drugs, neuromodulators, opioids, muscle relaxants, and cannabinoids [36-38]. Despite numerous pharmacological interventions available, monotherapy has not been found to be significant in successfully treating chronic pelvic pain, pointing to a multimodal approach for treatment [39-41]. This approach combines both pharmacological agents and nonpharmacological therapy (eg, bladder training, mindfulness) [42,43]. One method of multimodal therapy is employing combination pharmacological therapy. With any one pharmacological agent, there are often significant adverse events that must be balanced in order to ensure patient compliance and safety [44]. Combination therapy has been proposed as an alternative to monotherapy to minimize the adverse events of any one pharmacological agent, while combining the efficacy of multiple agents.

### Why It Is Important to Perform This Review

Although conditions producing chronic pelvic pain may occur separately in patients, chronic pain disorders are often found to co-occur in patients, leading to the NIH creating the term chronic overlapping pain conditions [45]. Clinical studies of chronic overlapping pain conditions have shown that comorbid pain conditions may exacerbate each other, and treatment of one pain syndrome may result in improvement of another as well [46,47]. As a result, it is worthwhile to investigate these disorders collectively when assessing the efficacy of potential treatments. It is important to investigate combination pharmacological therapy, as currently there are a great number of treatment options available for clinicians, each with a different efficacy and safety profile. Such a wide array of treatments can pose a challenge for clinicians trying to find the most evidence-based treatments for their patients in an individualized manner. Although it is recognized that there are major differences between genders in etiology, presenting phenotype, and outcomes in various types of CPP, we wanted to examine conditions affecting all genders (such as interstitial cystitis) and different gender-specific disease processes (eg, scrotal contents pain in males versus endometriosis in females) and determine similarities and differences in therapeutic efficacy of the multimedical therapies in those of different genders. By including both males and females in our review, we can ensure that evidence in this area is not missed. This systematic review seeks to synthesize the available data for all combination pharmacological therapies and facilitate clinicians in this decision-making process. Thus, we aim to conduct a systematic review to evaluate the efficacy and safety of combination pharmacological therapy for treating chronic pelvic pain.

### Objective

The objective of this review is to evaluate available clinical studies describing the efficacy and safety of combination drug therapies for the treatment of chronic pelvic pain.

## Methods

### Study Registration

This protocol is developed in accordance with PRISMA-P (Preferred Reporting Items for Systematic Reviews and Meta-Analyses Protocols) guidelines [48] (Multimedia Appendix 1) and PRISMA Harms guidelines [49] and is registered in the PROSPERO (International Prospective Register of Systematic Reviews) register (registration number CRD42020192231).

### Types of Studies

This review will include double-blind, randomized, controlled clinical trials (RCTs) of combination drug therapies in the treatment of chronic pelvic pain of any etiology—with an outcome measure of pain intensity or pain relief, assessed by a validated measurement tool. Included trials will compare combinations of two or more different drugs to at least one of the combination’s single agent components for the treatment of chronic pelvic pain. RCTs with less than 10 participants will be excluded to minimize study bias. Only English-language studies will be included.

### Types of Participants

We will include studies involving women, men, or both, aged 18 years and over, who report CPP of any etiology lasting for at least three months. The diagnosis of these conditions may have been made by a medical specialist, or a medical diagnosis made as part of the study procedures. We will exclude trials of primary or secondary dysmenorrhea.

### Types of Interventions

We will include any combination of two or more drugs administered systemically by any route of administration. We will include studies comparing combination drug therapy with at least one of the combination’s single agent components and also possibly placebo, or other pharmacological interventions.

### Types of Outcome Measures

The primary outcome for this review will be pain intensity or pain relief, assessed by a validated measurement tool. Secondary outcomes will include adverse events, serious adverse events, sexual dysfunction, health-related quality of life, depression and anxiety, and urinary symptoms.

### Search Methods for Identification of Studies

We will conduct a detailed search for clinical studies using CENTRAL, MEDLINE, and EMBASE from the dates of their establishment to the time the searches are conducted. We will search the clinical trial registry platform ClinicalTrials.gov to identify unpublished RCTs. We will also review the bibliographies of any clinical studies identified for relevance to identify additional published or unpublished data. The search strategy was developed in consultation with a librarian specializing in literature searches (Multimedia Appendix 2).

### Data Collection and Analysis

**Figure 1:**
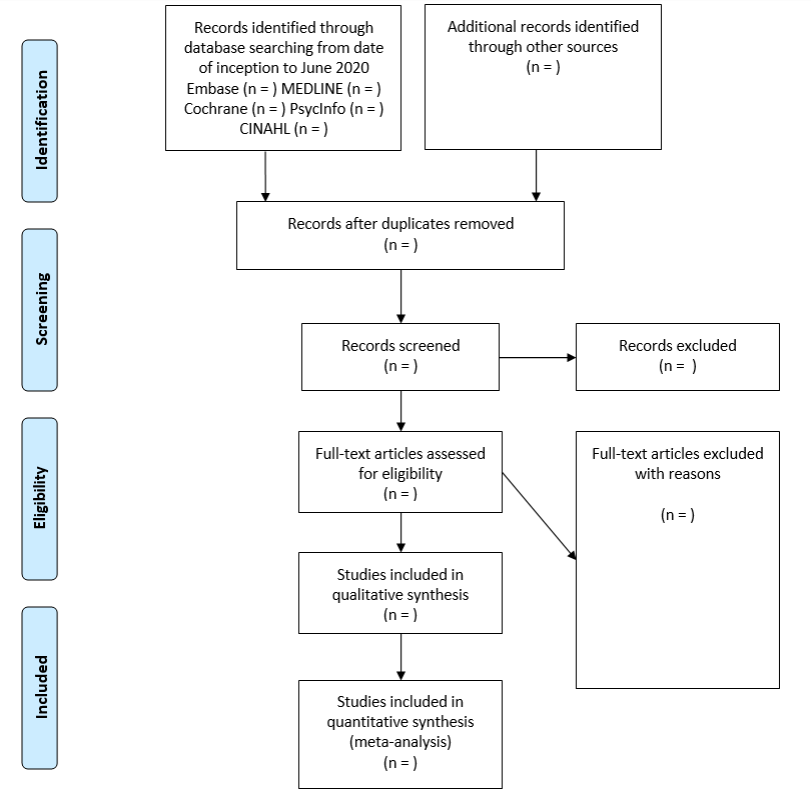
PRISMA flow diagram of study selection. PRISMA: Preferred Reporting Items for Systematic Reviews and Meta-Analyses.

Two reviewers (MM and RP) will independently evaluate studies for eligibility using the Covidence systematic review platform. Screening will be performed on titles and abstracts, thereafter full-text screening will be performed on citations that are determined to meet inclusion criteria. Reasons for study exclusion will be recorded. Any disagreements between the two reviewers will be resolved by discussion and consensus; if necessary, a third reviewer (IG) will be consulted. The planned selection process is shown in a PRISMA-P flowchart (Figure 1). Data extraction from included studies will be performed independently by two reviewers using predesigned data extraction forms. Forms will be piloted and revised if necessary. They will include the following variables:

- Publication details: first author’s name, publication year, country, language
- Study design: participant inclusion and exclusion criteria, randomization method, blinding method and assessment, number of participants by study and intervention arm, type of control, follow-up time, attrition rate, use of other treatments
- Participant characteristics: demographic, sample size, average age, sex distribution, presence of pain condition leading to chronic pelvic pain (such as chronic prostatitis), duration of chronic pelvic pain, comorbid pain conditions, presence of psychiatric conditions, baseline pain scores, baseline secondary outcome scores (sleep, quality of life, etc)
- Intervention and comparator details: details of pharmacological agents used (dose, route, frequency, duration), comparator used and details (placebo, active pharmacological agent)
- Outcome details: method of pain assessment (validated pain scale, etc), mean and standard deviation of pain scores and all continuous secondary outcomes, assessment timepoints, frequencies of adverse events and serious adverse events, method of adverse event assessment
- Other details: dropout rate with reasons, funding sources, conflicts of interest

Study authors will be contacted as required to request missing or incomplete data, and to clarify methods or findings.

### Assessment of Risk of Bias

Risk of bias for each study included in the review will be independently assessed by two reviewers using the criteria outlined in the Cochrane Handbook for Systematic Review of Interventions [50]. Disagreements between reviewers will be resolved through discussion and consensus and—if necessary—a third reviewer (IG) will be consulted. The following data on risk of bias will be assessed for each study: (1) random sequence generation (selection bias), (2) allocation concealment (selection bias), (3) blinding of participants and study personnel (performance bias), (4) blinding of outcome assessment (detection bias), (5) incomplete outcome data (attrition bias), (6) selective reporting (reporting bias), and (7) other potential sources of bias. Each domain will be judged as “low risk,” “high risk,” or “unclear risk” of bias, and individual bias items will be evaluated as described in the Cochrane Handbook for Systematic Reviews of Interventions [50].

### Measures of Treatment Effect

We expect that there will be a limited scope for meta-analysis because of the range of different outcomes measured across the small number of existing trials. However, where studies have used the same combination and comparator, with the same outcome measure, we will pool the results using a random-effect meta-analysis with an inverse variance method. We plan to calculate mean differences for continuous outcomes and, if needed, the standardized mean differences. If we employ standardized mean differences, we plan to transform these scores to a 0-10 scale to obtain the mean difference. If a meta-analysis if not feasible, we will provide a narrative synthesis of the findings from the included studies, structured around the type of intervention, target population characteristics, and outcome. A descriptive approach will be used to evaluate how combination drug therapies differ from monotherapy or other comparators, in managing other features of chronic pelvic pain such as anxiety, depression, quality of life, and functional disability. Any comparisons of multiple drug combinations and other placebo or active comparators will also be analyzed. In studies where more than one active treatment group is present, the control treatment group will be divided among the active treatment arms. The software RevMan (version 5.3; The Cochrane Collaboration, The Nordic Cochrane Centre) will be used for analysis. Heterogeneity between the studies within a meta-analysis will be assessed using the I^2^ statistic if the number of included studies is >10, in keeping with Cochrane best practices [50]. There are no pre-specified subgroup analyses planned at this time.

### Ethics and Dissemination

This review does not require ethical approval because no personal data collection is involved. The results of this systematic review will be published in a peer-reviewed journal.

## Results

To date, the protocol has been registered in PROSPERO and the search strategy has been finalized for starting data screening. Results are expected in September 2021.

## Discussion

Chronic pelvic pain syndromes have a significant prevalence among males and females, and the wide variety of etiologies for them creates a challenge for clinicians in their management. It has significant negative impact on patients’ quality of life, functioning, productivity in employment, relationships, and sexual function, which all contribute to the significant economic burden on the health care system [2,3,51]. As seen with the recent opioid crisis, management of chronic pain without evidence-based guidelines can have profound effects on patients’ morbidity and mortality rates, with deaths in the United States alone quadrupling over the last 15 years [52]. In a similar fashion, the overprescribing of certain classes of pharmacological therapies for chronic pelvic pain may lead to significant adverse events, a reduction in patient compliance, and a trajectory of persistent pain.

This review seeks to synthesize the evidence of efficacy and safety behind the combination of pharmacological agents in managing chronic pelvic pain. This synthesis will provide health care professionals with information on which pharmacological agents, and in what combination, are the most efficacious for certain etiologies of chronic pelvic pain (eg, IC/BPS vs CP/CPPS and CPP). Additionally, this review seeks to provide information on the safety and potential adverse events associated with pharmacological agents. This information can aid clinicians in tailoring a treatment approach for a specific patient and counselling them appropriately on both therapeutic benefit and side effects to better manage expectations and promote adherence to treatment.

## Appendix

Multimedia Appendix 1 PRISMA-P (Preferred Reporting Items for Systematic Reviews and Meta-Analyses Protocols) checklist.

Multimedia Appendix 2 Search strategy developed for EMBASE.

## Abbreviations

AE: adverse event
CIHR: Canadian Institutes of Health Research
CP/CPPS: chronic prostatitis/chronic pelvic pain syndrome
CPP: chronic pelvic pain
IC/BPS: interstitial cystitis/bladder pain syndrome
LUTS: lower urinary tract symptoms
NIH: National Institutes of Health
PRISMA-P: Preferred Reporting Items for Systematic Reviews and Meta-Analyses Protocols
PROSPERO: International Prospective Register of Systematic Reviews
RCT: randomized controlled trial
