# Supplementary material for: Efficacy and Safety of Drug Combinations for Chronic Pelvic Pain: Protocol for a Systematic Review
Source: JMIR Res Protoc. 2021 May 17;10(5):e21909. doi: 10.2196/21909 (PMC8167620; doi:10.2196/21909)
Supplement: Multimedia Appendix 2 [file resprot_v10i5e21909_app2.docx]

**Table 1. Search strategy for EMBASE.**

| 1 urethral pain syndrome.mp. (16)  2 urethral pain/ (370)  3 prostate pain.mp. (36)  4 exp scrotal pain/ or scrotal pain syndrome.mp. (2693)  5 penile pain.mp. (509)  6 testicular pain.mp. (905)  7 epididymal pain.mp. (23)  8 post-vasectomy pain.mp. (52)  9 exp cystitis/ (26492)  10 exp prostatitis/ (11075)  11 exp urethritis/ (8600)  12 epididymorchitis.mp. (21)  13 pelvic floor muscle pain.mp. (10)  14 endometriosis associated pain.mp. (290)  15 vaginal pain.mp. or vagina pain/ (1183)  16 vulvar pain.mp. (616)  17 vestibular pain.mp. (69)  18 clitoral pain.mp. (49)  19 proctitis/ (6813)  20 anus fissure/ (3999)  21 hemorrhoid/ (9451)  22 pudendal pain.mp. (21)  23 pudendal neuropathy.mp. or pudendal neuralgia/ (462)  24 sacral spinal cord/ (3081)  25 pelvis pain syndrome/ (12777)  26 chronic prostatitis/ (2629)  27 bladder pain syndrome.mp. (1455)  28 chronic pelvic pain syndrome.mp. (1604)  29 or/1-28 (81555)  30 combination therap*.mp. (81298)  31 comparative effectiveness.mp. or comparative effectiveness/ (85933)  32 combin*.mp. (3080758)  33 multimodal.mp. (51312)  34 or/30-33 (3185358)  35 exp beta adrenergic receptor blocking agent/ (302005)  36 exp quinolone derivative/ (168864)  37 exp tetracycline derivative/ (181288)  38 phytotherapy/ (17588)  39 exp nonsteroid antiinflammatory agent/ (753813)  40 allopurinol/ (23511)  41 botulinum toxin A/ (21929)  42 exp steroid 5alpha reductase inhibitor/ (11238)  43 doxazosin/ (7171)  44 levofloxacin/ (35754)  45 terazosin/ (3556)  46 exp muscle relaxant agent/ (158765)  47 dipyrone/ (8662)  48 exp muscarinic receptor blocking agent/ (126158)  49 palmidrol/ (1258)  50 thioctic acid/ (9054)  51 Sabal/ (1060)  52 Sabal extract/ (923)  53 selenium/ (40111)  54 lycopene/ (6952)  55 bromelain/ (2443)  56 exp ascorbic acid/ (103754)  57 dimethyl sulfone/ (640)  58 thiocolchicoside/ (414)  59 exp alpha adrenergic receptor blocking agent/ (318336)  60 cyclosporine/ (10726)  61 exp corticosteroid/ (1017360)  62 pollen extract/ (1942)  63 exp Calendula/ (662)  64 exp curcuma/ (4243)  65 quercetin/ (30663)  66 bioflavonoid/ (1177)  67 exp cholinergic receptor blocking agent/ (242111)  68 exp antidepressant agent/ (442583)  69 exp anticonvulsive agent/ (420728)  70 mepartricin/ (283)  71 om 89/ (153)  72 escherichia coli extract.mp. (103)  73 pentosan polysulfate/ (2190)  74 exp phosphodiesterase inhibitor/ (74019)  75 tadalafil/ (6835)  76 tanezumab/ (326)  77 exp oral contraceptive agent/ (69679)  78 exp gestagen/ (191510)  79 danazol/ (8624)  80 exp gonadorelin agonist/ (16157)  81 cimetidine/ (32175)  82 hydroxyzine/ (9158)  83 hyaluronic acid/ (43212)  84 chondroitin sulfate/ (13030)  85 resiniferatoxin/ (1695)  86 paracetamol/ (90545)  87 mycobacterium bovis bcg/ (1973)  88 dimethyl sulfoxide/ve [Intravesical Drug Administration] (183)  89 heparin/ve [Intravesical Drug Administration] (128)  90 pentosan polysulfate/ve [Intravesical Drug Administration] (61)  91 oxybutynin/ve [Intravesical Drug Administration] (237)  92 exp antibiotic agent/ (1562942)  93 exp serotonin noradrenalin reuptake inhibitor/ (173783)  94 serotonin 3 antagonist/ (3877)  95 ndri.mp. (167)  96 exp local anesthetic agent/ (256205)  97 or/35-96 (4683355)  98 29 and 34 and 97 (7780)  99 limit 98 to english language (6995) |
| --- |
